# The distribution of antibiotic resistance genes in chicken gut microbiota commensals

**DOI:** 10.1038/s41598-021-82640-3

**Published:** 2021-02-08

**Authors:** Helena Juricova, Jitka Matiasovicova, Tereza Kubasova, Darina Cejkova, Ivan Rychlik

**Affiliations:** 1 Veterinary Research Institute, Brno, Czech Republic; 2 Department of Biomedical Engineering, Brno University of Technology, Brno, Czech Republic

**Keywords:** Microbiology, Molecular biology

## Abstract

Antibiotic resistance in bacterial pathogens or several indicator bacteria is commonly studied but the extent of antibiotic resistance in bacterial commensals colonising the intestinal tract is essentially unknown. In this study, we aimed to investigate the presence of horizontally acquired antibiotic resistance genes among chicken gut microbiota members in 259 isolates with known whole genomic sequences. Altogether 124 isolates contained at least one gene coding for antibiotic resistance. Genes coding for the resistance to tetracyclines (detected in 101 isolates), macrolide-lincosamide-streptogramin B antibiotics (28 isolates) and aminoglycosides (25 isolates) were the most common. The most frequent tetracycline resistance genes were *tet*(W), *tet*(32), *tet*(O) and *tet*(Q). *Lachnospiraceae* and *Ruminococcaceae* frequently encoded *tet*(W). *Lachnospiraceae* commonly coded also for *tet*(32) and *tet*(O). The *tet*(44) gene was associated with *Erysipelotrichaceae* and *tet*(Q) was detected in the genomes of *Bacteroidaceae* and *Porphyromonadaceae*. Without any bias we have shown that antibiotic resistance is quite common in gut commensals. However, a comparison of codon usage showed that the above-mentioned families represent the most common current reservoirs but probably not the original host of the detected resistances.

## Introduction

The spread of antibiotic resistance via horizontal gene transfer belongs among one of the most serious challenges in current medicine. Due to increasing resistance to antibiotics in pathogens like *Klebsiella*, *Staphylococcus*, *Pseudomonas* or *Salmonella*^1,2^, diseases caused by these pathogens, although possible to control when caused by antibiotic-sensitive strains, become serious threats when caused by antibiotic-resistant clones. Antibiotics are not strictly selective against only pathogens, thus whenever antibiotics are used, not only the target pathogen but also commensal microbiota is affected and subjected to selection for resistant clones. Any use of antibiotics to control infection caused by a single bacterial pathogen therefore inevitably leads to the selection of hundreds of commensal species resistant to their action as well. The resistant commensals may later act as reservoirs of antibiotic resistance genes, quite extensive reservoirs, since microbiota of distal parts of intestinal tract consists of approx. one thousand different species with a population density of around 10^10^ bacterial cells per gram of digesta.

Studies on the distribution of antibiotic resistance among gut microbiota are limited due to their specific culture requirements. This is the reason why antibiotic resistance of gut colonisers has been mainly studied in *E. coli*, lactobacilli and bifidobacteria for which selective culture conditions are known^3–5^. Unfortunately, specific and selective culture conditions are not known for other gut microbiota members and information on the distribution of antibiotic resistance in *Bacteroides*, *Parabacteroides*, *Faecalibacterium*, *Butyricicoccus*, *Blautia* or *Sutterella* etc. is much more limited.

Since selective culture conditions for the majority of gut microbiota are not known, alternative protocols for characterisation of the gut microbiota resistome have been used. One of the most frequently used protocols includes shotgun sequencing of DNA purified from faecal material or intestinal digesta. Those performed in chickens demonstrated that chicken gut microbiota represents an important source of antibiotic resistance genes with the most abundant genes encoding different drug efflux pumps, resistances to fluoroquinolones and tetracyclines^6–8^.

Despite shedding light on total ARG content, metagenomic sequencing fails to determine the original bacterial host of the detected antibiotic resistance since some of the antibiotic resistance genes spread as a single gene cassette^9^. Following shotgun sequencing and metagenomic assembly, such cassettes form separate contigs, which are impossible to associate with the rest of the host bacterial genome. Antibiotic resistance genes are also commonly part of mobile genetic elements that can be present in a wide variety of organisms^10,11^. The detection of such contigs after metagenomic assembly, e.g. a plasmid DNA sequence, again is unable to determine in which bacterial species such a plasmid was present.

The above-mentioned limits can be overcome by culture of gut anaerobes followed by whole genome sequencing. Although anaerobic culture still represents a limiting step, significant advances in the culture of gut anaerobes have been reported recently^12–15^. Since we have cultured and sequenced hundreds of chicken gut microbiota members^15,16^, in this study we searched their genomic sequences for the presence of antibiotic resistance genes. This enabled us to address questions like (i) which antibiotic resistance genes are the most widespread among chicken gut microbiota, (ii) which taxa behave as the most important reservoirs of antibiotic resistance among gut commensals, (iii) which genes are tightly associated with a limited number of taxa and (iv) which genes can be spread among distantly related bacterial species.

## Results

### Bacterial strains and identification of acquired resistance genes

Altogether 259 bacterial isolates obtained from the chicken caeca in pure culture were included in this study. The whole genome sequences were determined and based on the 16S rDNA sequences, the isolates were classified to eight different phyla; *Firmicutes* (159 isolates), *Bacteroidetes* (50 isolates), *Actinobacteria* (38 isolates), *Proteobacteria* (6 isolates), *Fusobacteria* (3 isolates), *Verrucomicrobia* (1 isolate), *Elusimicrobia* (1 isolate) and *Synergistetes* (1 isolate).

**Figure 1:**
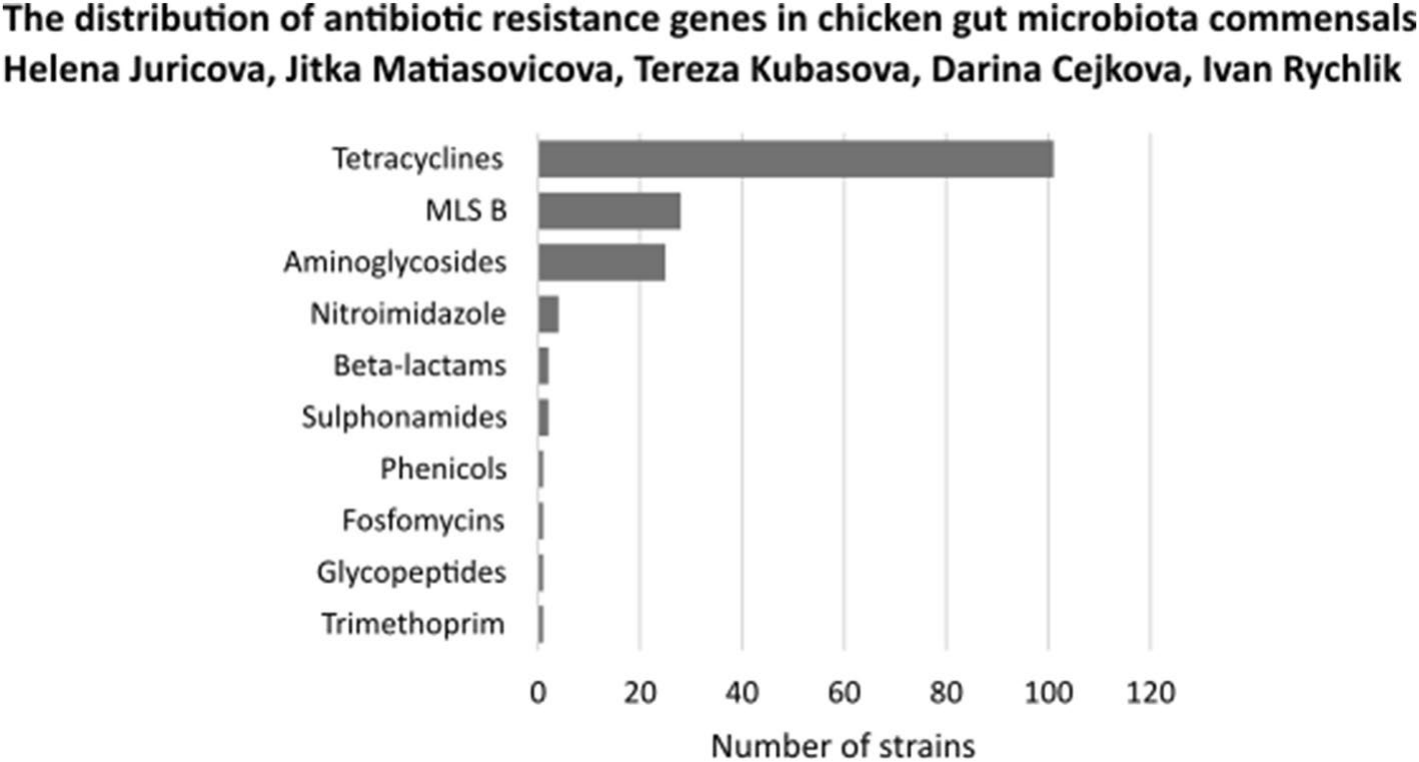
Acquired antibiotic resistance genes in chicken gut anaerobes. Whole genomic sequences of 259 gut anaerobes were searched using ResFinder for the presence of horizontally acquired genes responsible for antibiotic resistance. See Supplementary Fig. S1 for the distribution of these resistances among individual isolates.

A comparison using the ResFinder database showed that 124 isolates (47.9% out of all) harboured at least one antibiotic resistance gene (Supplementary Fig. S1). Resistance genes coding for a single antibiotic were detected in 87 isolates. Genes responsible for resistance to two different antibiotics were detected in 32 isolates and additional 5 isolates encoded genes responsible for resistance to three different antibiotics. The recorded genes coded for resistance to tetracyclines (detected in 101 isolates), macrolide-lincosamide-streptogramin B antibiotics (28 isolates), aminoglycosides (25 isolates), nitroimidazole (4 isolates), β-lactams (2 isolates), sulphonamides (2 isolates), and phenicols, fosfomycins, glycopeptides, and trimethoprim resistance, each found in a single isolate (Fig. 1). In 135 isolates (52.1% out of all), no gene encoding antibiotic resistance was identified using ResFinder database. Low frequency of antibiotic resistance genes was recorded mainly in the isolates belonging to phylum *Actinobacteria* and to family *Veillonellaceae* (Supplementary Fig. S1).

### Identification of tetracycline resistance genes

Since genes coding for resistance to tetracyclines were the most common, we analysed their distribution in greater detail. Due to the fact that some of the isolates harboured more than one gene responsible for tetracycline resistance, altogether 114 tetracycline resistance genes were found in 101 isolates. Three of them, *tetA*(P), *tet*(L) and *tet*(40), encoded efflux pump proteins, eight of them, *tet*(M), *tet*(S), *tet*(44), *tet*(O), *tet*(32), *tet*(Q), *tet*(W) and *tetB*(P) encoded ribosomal protection proteins (RPP) and one mosaic gene *tet*(O/32/O) of RPP type of resistance was also recorded (Table 1).

**Table 1:** Tetracycline resistance genes and their association with isolates belonging to particular families of chicken gut microbiota.

Family	n strains	Genomic GC%	*tet*Q	*tet*W	*tet*32	*tet*O/32/O	*tet*40	*tet*O	*tet*M	*tet*L	*tet*S	*tetB*P	*tet*44	*tetA*P
GC% tet genes			40.0	53.2	42.8	41.3	41.2	40.5	36.0	35.3	33.4	31.8	30.4	29.6
Rikenellaceae	5	60.8	1	–	–	–	–	–	–	–	–	–	–	–
Porphyromonadaceae	10	48.2	4	–	–	–	–	–	–	–	–	–	–	–
Bacteroidaceae	33	46.1	9	–	–	–	–	–	–	–	–	–	–	–
Coriobacteriaceae	32	66.3	–	5	–	–	–	–	–	–	–	–	–	–
Ruminococcaceae	55	59.5	–	34	2	1	–	–	–	–	–	–	–	–
Lachnospiraceae	34	49.1	–	15	8	–	1	8	–	–	–	–	–	–
Veillonellaceae	11	41.6	–	1	–	–	–	–	–	–	–	–	–	–
Enterococcaceae	4	38.2	–	–	–	–	–	–	1	1	1	–	–	–
Lactobacillaceae	24	38.1	–	6	–	–	–	–	1	–	–	–	–	–
Erysipelotrichaceae	23	33.9	–	–	3	–	–	1	1	–	–	–	3	–
Clostridiaceae	5	30.1	–	–	–	–	–	–	–	–	–	3	–	4

### Taxonomic distribution of tetracycline resistance genes

**Figure 2:**
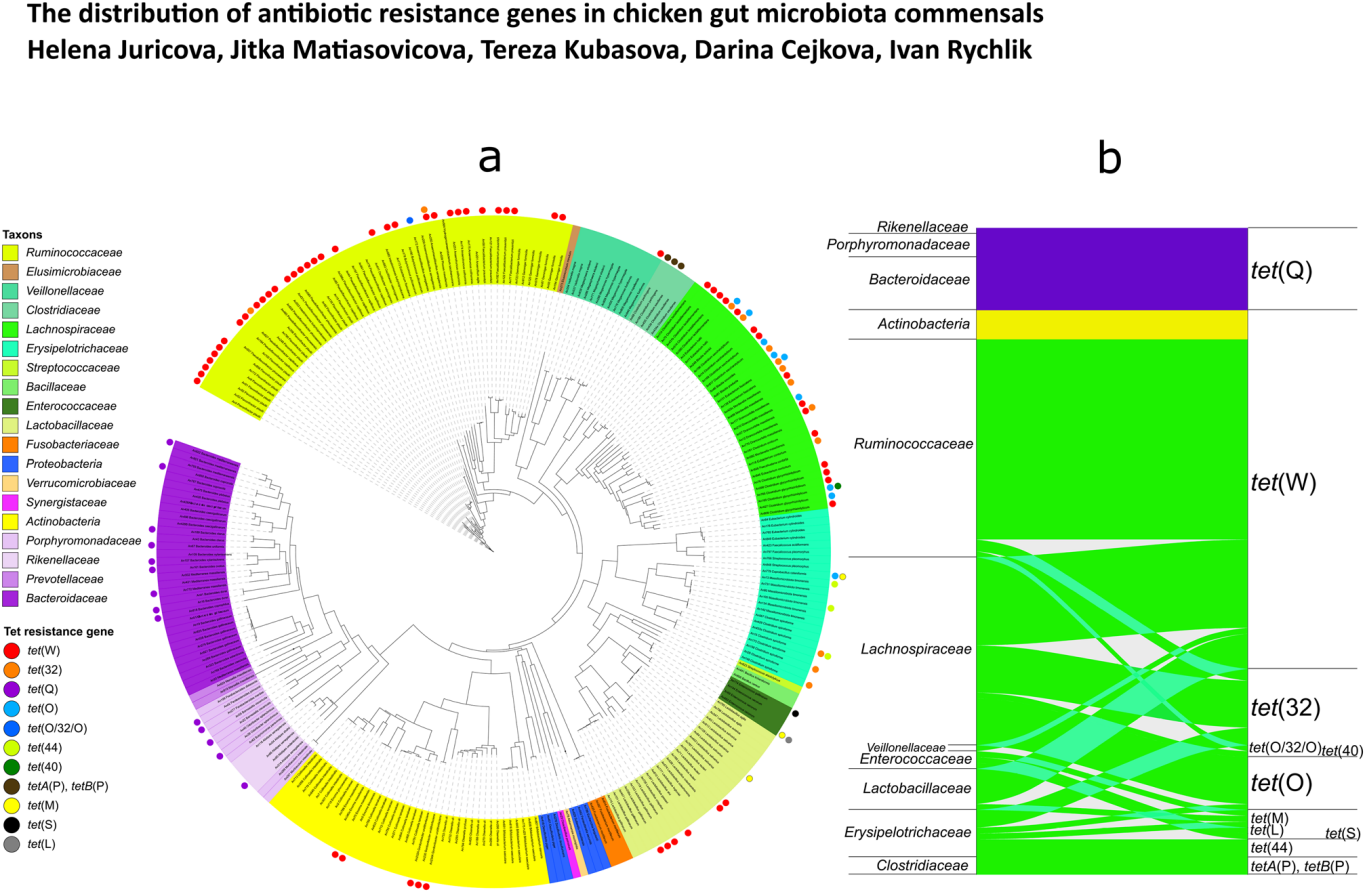
Presence of genes coding for resistance to tetracycline in isolates belonging to different phyla and families. (**a**) All isolates included in this study were aligned according to their 16S rRNA gene sequence. The phylogenetic tree was performed in iTOL v5.5.1, http://itol.embl.de^17^. The presence of particular tetracycline resistance genes is indicated by dots external to the dendrogram. (**b**) Distribution of tetracycline resistance genes among different families of chicken gut anaerobes. The alluvial diagram was produced in R v4.0.2, https://www.R-project.org/^18^.

Tetracycline resistance genes were detected among isolates belonging to three different phyla: *Firmicutes* (82 isolates with tetracycline resistance genes, i.e. 51.6% of all isolates from this phylum); *Bacteroidetes* (14 isolates, 28%); and *Actinobacteria* (5 isolates, 13.2%). In isolates belonging to the remaining phyla, no genes coding for tetracycline resistance were identified (Fig. 2a).

Some of the tetracycline resistance genes were restricted to a certain taxonomic unit. The *tet*(Q) gene was present only in isolates from phylum *Bacteroidetes* and *tetA*(P) was associated exclusively with family *Clostridiaceae*. The opposite extreme was represented by the *tet*(W) gene, which was detected in isolates belonging to 5 different families and two phyla. By analysing the same data set from a taxonomic perspective, *Actinobacteria* harboured only the *tet*(W) gene and *Bacteroidetes* encoded only *tet*(Q). On the other hand, high variability in tetracycline resistance genes was found among isolates from *Firmicutes* and families *Lachnospiraceae* and *Erysipelotrichaceae*, each of them encoding 4 different *tet* genes (Table 1, Fig. 2).

### Real-time PCR quantification of tetracycline resistance genes in complete chicken microbiota

Since the frequency of different tetracycline resistance genes and their association with particular taxa was determined in a rather small number of genomes, which could affect conclusions, we subsequently verified the frequency of distribution and association of the major taxa harbouring selected tetracycline resistance genes in 70 chicken caecal samples. In these samples, the abundance of *tet*(W), *tet*(32), *tet*(Q), *tet*(O), *tet*(44) and *tetA*(P) genes was determined by real-time PCR and microbiota composition was determined by 16S rRNA sequencing.

**Figure 3:**
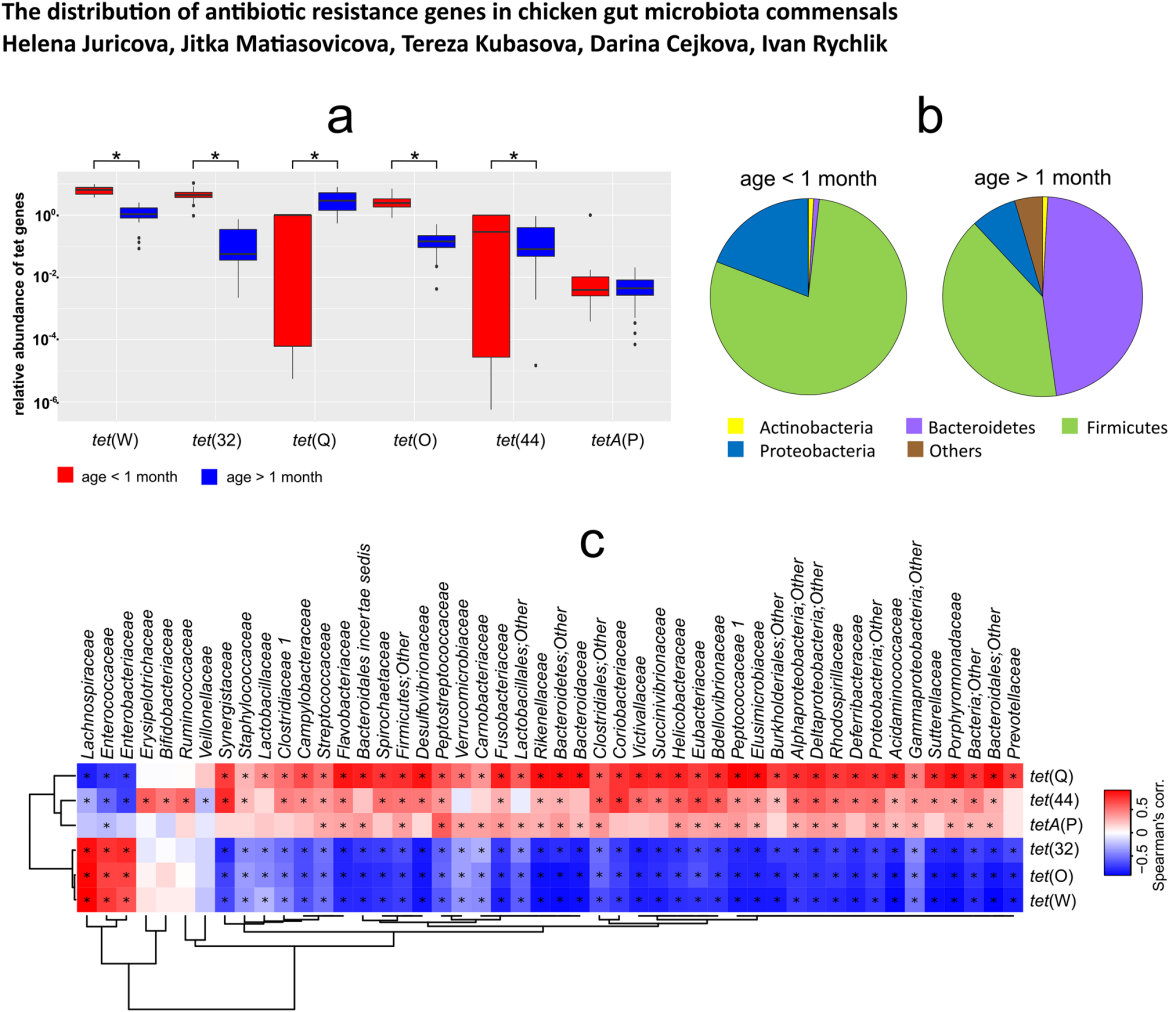
(**a**) Abundance of selected tetracycline resistance genes and (**b**) microbiota composition in chickens younger or older than 1 month. *, *p* < 0.05. In agreement with predictions from genomic analysis (Table 1 and Fig. 2), microbiota of chickens under 1 month of age was dominated by *Firmicutes* and *tet* genes characteristic for *Firmicutes* were commonly detected in these samples. On the other hand, *Bacteroidetes* formed approx. 47% of total microbiota of adult hens and *tet*(Q) gene, predicted as associated with *Bacteroidetes*, was significantly more abundant in the samples from adult chickens in comparison with the chickens younger than 1 month. (**c**) Correlation heat map of microbiota composition at family level and frequency of selected genes coding for tetracycline resistance. *, *p* < 0.05. The heat map was produced in R v4.0.2, https://www.R-project.org/^18^.

Among caecal samples originating from chickens younger than one month of age, *tet*(W), *tet*(32) and *tet*(O) genes were the most abundant tetracycline resistance genes with an abundance ranging from 1 to 5% of the bacterial population. The *tet*(44) and *tet*(Q) genes were present at an abundance of 0.01 to 0.1% and *tetA*(P) was the least abundant tetracycline resistance gene in microbiota of chickens younger than 1 month with an abundance of around 0.001% (Fig. 3a). On the other hand, when caecal samples originating from chickens older than 1 month were tested, the *tet*(Q) gene was the most frequently detected gene with an abundance ranging from 1 to 5%. The *tet*(W) gene was present at an abundance of 0.5 to 1% followed by *tet*(32), *tet*(44) and *tet*(O) that were present at an abundance of around 0.1%. The *tetA*(P) gene was the least abundant tetracycline resistance gene in microbiota of chickens older than 1 month with an abundance below 0.001% (Fig. 3a).

When the microbiota composition was determined in the same samples, those originating from chickens under 1 month of age were dominated by *Firmicutes* (79.0 ± 8.65%) and *Proteobacteria* (19.14 ± 7.81%). *Bacteroidetes* and *Actinobacteria* were present in less than 1% of total microbiota (Fig. 3b). On the other hand, caecal samples originating from chickens older than 1 month were dominated by *Bacteroidetes* and *Firmicutes*, which were present at 46.96 ± 18.79% and 40.28 ± 21.19%, respectively. *Proteobacteria* formed 7.4 ± 6.59% of the total bacterial population and *Actinobacteria* formed less than 1% of total microbiota (Fig. 3b). Correlation analysis confirmed predictions from genomic analyses and indicated that *Lachnospiraceae* currently represents the most likely reservoir of *tet*(W), *tet*(32) and *tet*(O), *Eubacteriaceae* and *Erysipelotrichaceae* represent the reservoir of *tet*(44), and *Bacteroidaceae*, *Porphyromonadaceae*, *Prevotellaceae* or *Flavobacteriaceae*, all from phylum *Bacteroidetes*, are the most likely reservoirs of *tet*(Q). The frequency of *tetA*(P) correlated the most with abundance of family *Peptostreptococcaceae* (Fig. 3c).

### Origin of tetracycline resistance genes

**Figure 4:**
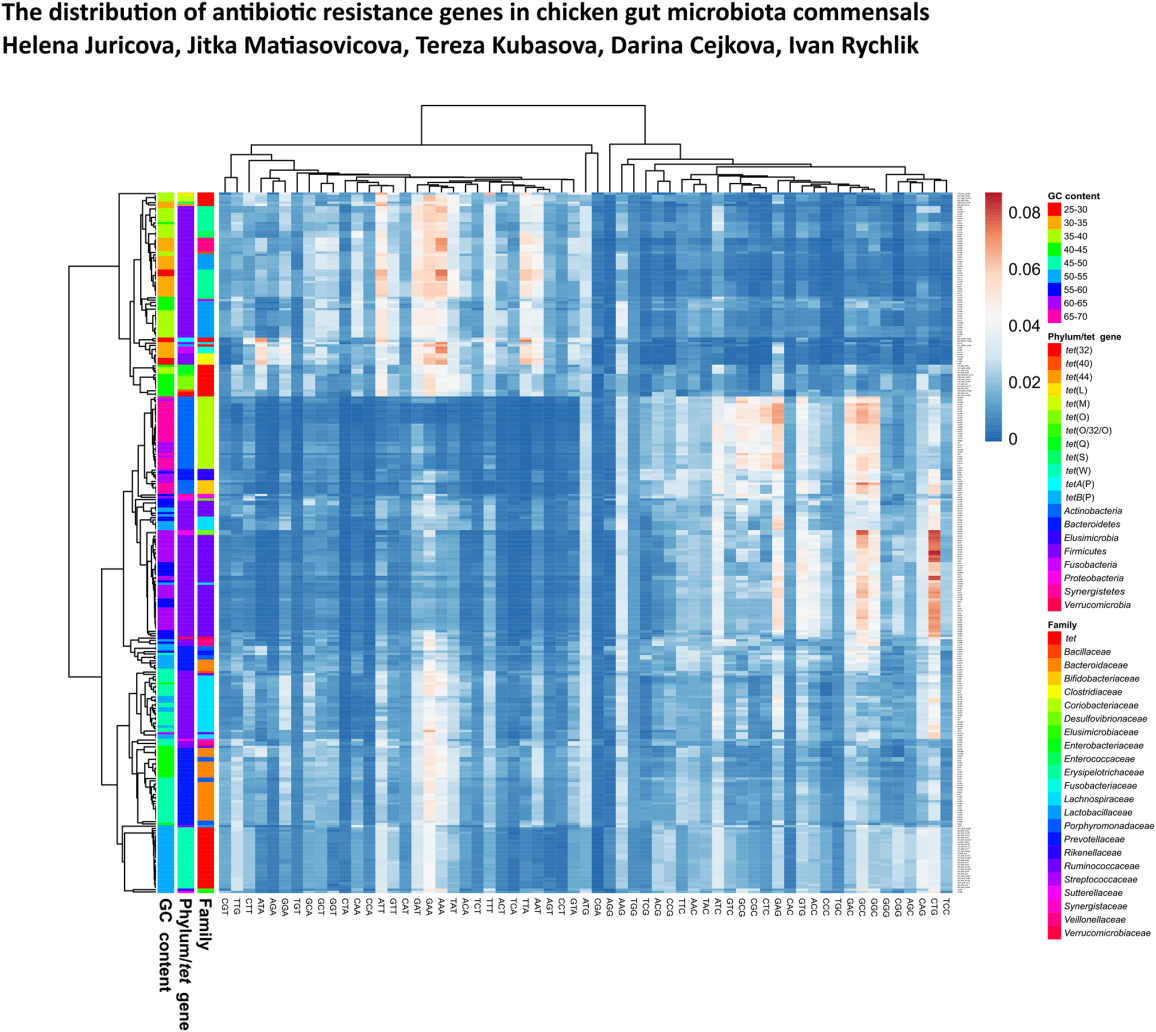
Clustering of all isolates analysed in this study and all tetracycline resistance genes based on GC content and codon usage. Frequency of codon usage in each of the tetracycline resistance genes and frequencies of codon usage averaged across all genes in the genomes of each of the analysed isolates were used for mutual clustering. Since *tet* proteins consisted of 408 to 657 amino acids, the distribution of each of the 61 codons coding for a particular amino acid could be considered as random and representative. However, since only a single stop codon may appear in each *tet* gene, frequency of stop codon usage was not considered. The heat map was constructed in ClustVis, https://biit.cs.ut.ee/clustvis/^19^.

Current reservoirs may not necessarily represent the original host. Finally, we therefore attempted to identify the most likely original source of each of the tetracycline resistance genes. To address this, GC content and codon usage in all *tet* resistance genes were compared with GC content and codon usage of genomes of analysed bacterial isolates (Table 1, Fig. 4). *tet*(W) gene had the highest GC content (53% GC content). The GC content of all the remaining tetracycline resistance genes ranged from 29 to 43%. According to hierarchical clustering of codon usage, *tet*(M), *tet*(S), *tet*(44), and *tet*(O), *tet*(32), *tet*(O/32/O), *tet*(40) formed two clusters of genes with similar codon usage, respectively, indicating their common origin. The *tet*(L) and *tet*(Q) genes each formed a separate lineage suggesting their independent origin. However, none of these genes clustered closely with any of the included genomes. On the other hand, *tetA*(P) and *tetB*(P) clustered together with *Tyzzerella* sp. An114 from family *Lachnospiraceae*, *Fusobacterium mortiferum*, *Clostridium saudiense* and *Clostridium perfringens* pointing to their potential original reservoir. When we assessed the origin of *tet* genes with bacteria at family level, except for *tet*(W), the origin of all tested *tet* genes could be traced to low GC content bacteria such as *Erysipelotrichaceae*, *Lactobacillaceae*, *Enterococcaceae*, *Peptostreptococcaceae* or *Clostridiaceae*.

## Discussion

In this study, we were interested in the dissemination of antibiotic resistance genes among chicken gut commensals, i.e. in bacterial species which have never been the target of antibiotic therapy. Our results show that nearly half of the 259 selected isolates possessed at least one of the horizontally acquired genes conferring resistance to antibiotics. In fact, the real number might be even higher because we could identify only the genes which were present in the ResFinder database. Even with this limit, genes coding for resistance to 10 different classes of antibiotics were recorded. Of these, genes coding for resistance to tetracyclines were the most common likely due to the broad use of tetracyclines as feed additives and growth promoters^20^.

Twelve different genes increasing resistance to tetracyclines were recorded with *tet*(W), *tet*(Q), *tet*(32) and *tet*(O) being the most frequent. This conclusion obtained after analysis of bacterial genomes was also confirmed by quantitative PCR in chicken caecal samples. The *tet*(W), *tet*(Q), *tet*(32) and *tet*(O) genes were identified among the most common tetracycline resistance genes also in pig manure^21^. The same genes were also found as common in human gut samples^22^. However, when performing such comparisons, it should be kept in mind that the frequency of a particular gene is affected by the sample type collected. We detected the *tet*(M) gene in *Lactobacillus* and *Enterococcus*. If we sampled ileal contents, which are rich in lactobacilli and enterococci^23^, we could expect a much broader *tet*(M) distribution. Similarly, if sampling faecal material, which mostly represents discharges of ileal digesta and less frequently caecal excretions^24^, we would also expect a much broader *tet*(M) prevalence as previously proposed^25^. Alternatively, if collecting samples from young chickens, e.g. broilers, the *tet*(Q) gene would be underrepresented since this gene is present in *Bacteroidetes*^26,27^ and isolates of this phylum usually appear in chicken gut microbiota later during life^28^.

The *tet*(W) gene has been previously reported as one of the most widespread tetracycline resistance genes, present in anaerobic bacteria from geographically distant locations^25^. The *tet*(W) gene has been found in *Clostridium* spp., *Roseburia* spp., *Selenomonas* spp., *Mitsuokella* spp., *Megasphaera elsdenii*, *Bifidobacterium longum* from bovine and sheep rumen, and porcine and human faeces^9,29^. The *tet*(W) gene can therefore spread among phyla (*Firmicutes* and *Actinobacteria*). Within *Firmicutes*, it can be found in *Veillonellaceae* (*Selenomonas*, *Mitsuokella*, and *Megamonas funiformis* in this study), which express an outer membrane similar to Gram-negative bacteria^30^. Perhaps not surprising, conserved sequences flanking *tet*(W) gene are quite short, only 657 bp upstream and 43 bp downstream of the *tet*(W) gene^9^. The genetic context of *tet*(W) therefore varies widely thus enabling this gene to successfully spread among distantly related bacteria. Despite this, *tet*(W) genes from different isolates formed a separate cluster in codon usage analysis and their common origin is likely.

The *tet*(32) gene was originally detected in two isolates of *Streptococcus* spp. and one isolate of *Eubacterium saburreum* from the oral cavity^31^. This agrees with our observation when we recorded this gene in genomes of *Lachnospiraceae*, *Ruminococcaceae*, and *Erysipelotrichaceae*, all common gut microbiota members from phylum *Firmicutes*.

We detected the *tet*(O) gene only in *Lachnospiraceae* and *Erysipelotrichaceae*. However, exactly the same gene has been reported also in *Campylobacter*^32,33^ and we have found the same gene also in *Fusobacterium mortiferum* and *Fusobacterium perfoetens* from pig gut microbiota (unpublished data). This makes the *tet*(O) gene phylogenetically the most widespread, capable of crossing the barrier between Gram-positive and Gram-negative bacteria.

Finally, we attempted to define current reservoirs and possible original sources of individual tetracycline resistance genes. We have shown recently that the addition of antibiotics into growth media positively selected for *Erysipelotrichaceae*^15^. In agreement, we found 4 different tetracycline resistance genes in the genomes of 23 isolates from family *Erysipelotrichaceae*. Additional important reservoirs should be sought in family *Lachnospiraceae* since 34 isolates from this family encoded 32 tetracycline resistance genes of 4 different types (*tet*(W), *tet*(32), *tet*(O/32/O), *tet*(40)). On the other hand, numerically the most represented family *Ruminococcaceae* (n = 55 in this study) encoded only 3 different tetracycline resistance genes. Differential distribution of tetracycline resistance genes among gut microbiota members can be explained by GC content and codon usage in tetracycline resistance genes and analysed genomes as can be seen in Table 1 in which tetracycline resistance genes and bacterial families are arranged according to descending GC content. In *Coriobacteriaceae* (66.3% average genomic GC content), only the *tet*(W) gene with the highest GC content of all *tet* genes (53.2% GC content) was detected, similar to a previous report^5^. In *Ruminococcaceae*, with 59.5% average genomic GC content, *tet*(W) with 53.2% GC content was the most frequent *tet* gene followed by *tet*(32) and recombinant *tet*(O/32/O) genes with 41–42% GC content. The *tet* genes with lower than 40% GC content were not detected in *Ruminococcaceae*. *Erysipelotrichaceae*, with an average genomic GC content of 33.9%, encoded only *tet* genes with a GC content between 30.4 and 40.4%. Finally, 5 strains of family *Clostridiaceae* with average genomic GC content 30.1% encoded only *tetA*(P) and *tetB*(P) with GC content 29.6 and 31.8%, respectively. This dependence was present also in *Bacteroidetes* since *tet*(Q) (40.0% GC content) was common in *Bacteroidaceae* (46.1% GC content) and *Porphyromonadaceae* (48.2% GC content) but rare in *Rikenellaceae* (60.8% GC content). Currently, *Lachnospiraceae* therefore represent the most important reservoir of *tet*(W), *tet*(32), *tet*(O/32/O), *tet*(40) and *tet*(O), *Erysipelotrichaceae* of *tet*(44), *Clostridiaceae* of *tetA*(P) and *tetB*(P), and *Bacteroidaceae*, and *Porphyromonadaceae* of *tet*(Q).

Although we were able to identify currently the most important reservoirs of the antibiotic resistance genes, predicting the original host of the tested *tet* genes was more difficult. Except for *tetA*(P) and *tetB*(P), all the remaining tetracycline resistance genes exhibited codon preferences different from the bacteria included in this study. The tested bacteria therefore do not represent the original source of tetracycline resistance and this resistance has been introduced to these species by horizontal gene transfer. Only *tetA*(P) and *tetB*(P) exhibited codon usage similar to *Tyzzerella* sp., *Fusobacterium mortiferum*, *Clostridium saudiense* and *Clostridium perfringens*. Any of these species may therefore represent an original source of this *tetA*(P) and *tetB*(P). The *tetA*(P) and *tetB*(P) genes are common to soil microbiota and Blau et al. reported an increased abundance of *tetA*(P) in manure-treated soil concurring with an enrichment of clostridia^34^. We therefore favour *Clostridium saudiense* and *Clostridium perfringens* and related low GC content clostridia as possible original source of this gene. However, we cannot exclude other species not included in this study as the original host of *tetA*(P) and *tetB*(P) since these genes were detected also in *Clostridium difficile*^35^ and we correlated its presence with *Peptostreptococcaceae*, i.e. the family to which *Clostridium difficile* belongs. The low GC content clostridia or *Peptostreptococcaceae* may represent the possible original source of *tetA*(P) and *tetB*(P).

## Methods

### Bacterial sample collection, whole genome sequencing and data availability

Altogether 259 isolates originating from chicken caecal contents were included in this study (Supplementary Table S1). The isolates were obtained from healthy chickens or hens as described previously^16^. DNA isolation, whole genome sequencing and bioinformatic analysis was performed according to Medvecky et al.^16^ and genomic sequences are deposited in NCBI under accession number PRJNA377666.

### Identification of acquired resistance genes

On-line version of the ResFinder available at https://cge.cbs.dtu.dk/services/ResFinder/ was used to identify acquired antibiotic resistance genes^36^. The threshold for a match between genes in the ResFinder database and the input genome sequence was set to 90% identity over 60% of the length of the resistance gene.

### GC content and codon usage

GC content and codon usage were calculated for all protein coding genes of all investigated genomes and all tetracycline resistance genes using CodonUsage python script embedded in BioPython^37^. All 3 stop codons were excluded from codon usage analysis since each antibiotic resistance gene uses only a single stop codon which does not allow for any variation.

### Real-time PCR quantification of tetracycline resistance genes in caecal samples

To verify in silico analyses and predictions, tetracycline resistance genes and microbiota composition were determined in 70 chicken caecal samples. Of these, 37 samples originated from chickens younger than 1 month of age and 33 samples originated from chickens older than 1 month (Supplementary Table S2). Classification into two age categories was adopted due to the known development of chicken caecal microbiota and the appearance of representatives of *Bacteroidetes* usually in chickens older than one month^28^. Caecal contents were homogenised in a MagNA Lyser (Roche) and the DNA was extracted using a QIAamp DNA Stool Mini Kit according to the manufacturer’s instructions (Qiagen). Purified DNA was used as a template for real-time PCR quantification of *tet*(W), *tet*(32), *tet*(Q), *tet*(O), *tet*(44) and *tetA*(P) tetracycline resistance genes (Table 2). Amplification of the 16S rRNA gene using *Eubacteria* specific primers was used as a reference to determine the total amount of eubacterial DNA in each sample. PCR reactions were performed in 3 μl volumes in 384-well microplates using QuantiTect SYBR Green PCR Master mix (Qiagen) as described previously^38^. After PCR, Ct values of genes of interest were subtracted from the Ct value of bacterial 16S rRNA gene amplification (ΔCt) and the relative abundance of each resistance gene was calculated as 2^-ΔCt^.

**Table 2:** List of primers used in this study.

Target gene	Forward primer 5′–3′	Reverse primer 5′–3′	Reference
*tet*(W)	AGCGACAGCGTGAGGTTAAA	AAGTTGCGTAAGAGCGTCCA	This study
*tet*(32)	GCTCACTCCGGAAGTGTCTC	TTCAAAGGTTCCCCCGCAAT	This study
*tet*(Q)	AGAATCTGCTGTTTGCCAGTG	CGGAGTGTCAATGATATTGCA	^39^
*tet*(O)	ACGGAAAGTTTATTGTATACC	TGGCGTATCTATAATGTTGAC	^39^
*tet*(44)	CGAAAGCAAAGTTTCACTCGGT	AAGCGAAAATCCGAGGGAGT	This study
*tetA*(P)	AGTTGCAGATGTGTACAGTCG	CTTCCGCAATCCAAGCTTCA	This study
16S rRNA	TCCTACGGGAGGCAGCAG	CGTATTACCGCGGCTGCT	This study

### Microbiota composition determined by 16S rDNA sequencing

The DNA extracted from caecal samples was used as a template in PCR with eubacterial primers amplifying the V3/V4 variable region of 16S rRNA genes. Following amplification, the products were processed exactly as described previously^23^. The amplicon data have been deposited in NCBI under accession number PRJNA673404.

### Bioinformatics and statistics

Clustal Omega using sequences of the whole gene for 16S rRNA was applied for clustering of all strains according to their taxonomic relatedness^40^. Final modification of the phylogenetic tree was performed in iTOL v5.5.1^17^.

Spearman’s correlation was used to calculate correlations between the abundance of a particular family in chicken gut microbiota and the abundance of selected tetracycline resistance genes. A final heat map as well as alluvial diagram were produced in R v4.0.2 using ggplot2 and gplots packages, respectively^18^.

Average relative frequencies of all codons in protein coding genes within an individual genome and relative frequencies of tetracycline resistance genes were used for heatmap construction and hierarchical clustering using ClustVis^19^. Data were clustered based on correlation distance and average linkage. For tetracycline resistance genes, only one representative from a group of identical genes (100% identity) was selected.

Differences in the distribution of antibiotic resistance genes in chickens younger or older than 1 month were determined using non-parametric Mann–Whitney U-test. Differences with *p* < 0.05 were considered as significant.

### Approval for animal experiments

Authors declare that not a single chicken has been sacrificed specifically for the purpose of this study and DNA purified from all chicken samples originated from previous studies. The handling of animals in these studies was performed in accordance with current Czech legislation (Animal Protection and Welfare Act No. 246/1992 Coll. of the Government of the Czech Republic) and the specific experiments were approved by the Ethics Committee of the Veterinary Research Institute followed by the Committee for Animal Welfare of the Ministry of Agriculture of the Czech Republic (permit number MZe1922).

## Supplementary Information

Supplementary Information.
