# Supplementary material for: The distribution of antibiotic resistance genes in chicken gut microbiota commensals
Source: Sci Rep. 2021 Feb 8;11:3290. doi: 10.1038/s41598-021-82640-3 (PMC7870933; doi:10.1038/s41598-021-82640-3)
Supplement: Supplementary file 1 — Supplementary Information. [file 41598_2021_82640_MOESM1_ESM.pdf]

The distribution of antibiotic resistance genes in chicken gut microbiota commensals

Helena Juricova, Jitka Matiasovicova, Tereza Kubasova, Darina Cejkova, Ivan Rychlik

SUPPLEMENTARY FILES

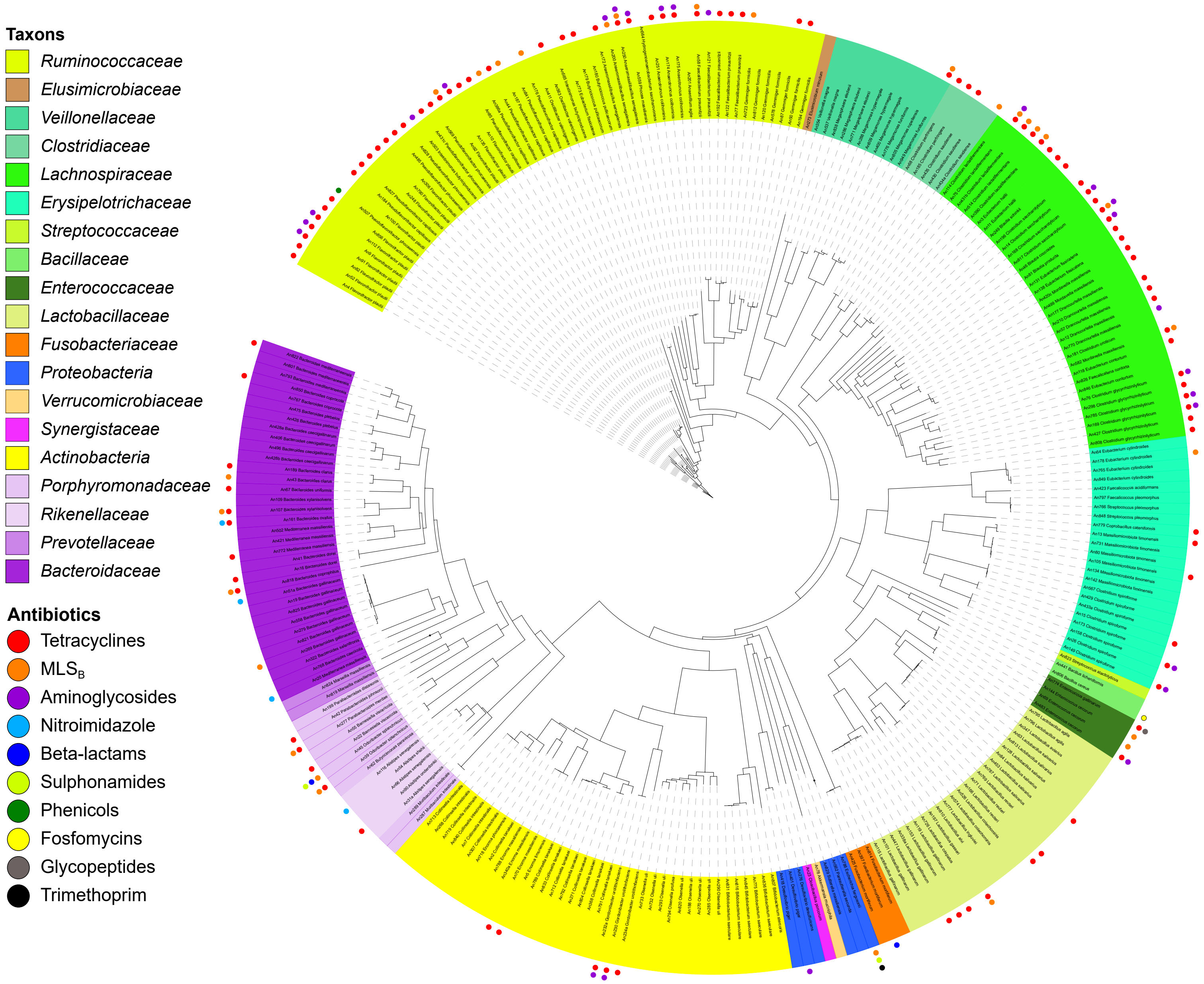

Supplementary Figure S1. Acquired antibiotic resistance genes in chicken gut anaerobes. All isolates were aligned according to their 16S rRNA sequence. The phylogenetic tree was performed in iTOL v5.5.1 (http://itol.embl.de). The presence of genes coding for resistance to different antibiotics is indicated by dots external to the dendrogram.

BioProject	BioSample	Sample Name	strain
PRJNA377666	SAMN06473570	Flavonifractor plautii An10	An10
PRJNA377666	SAMN06473571	Flavonifractor plautii An100	An100
PRJNA377666	SAMN06473572	Lactobacillus gallinarum An101	An101
PRJNA377666	SAMN06473576	Massiliomicrobiota timonensis An105	An105
PRJNA377666	SAMN06473578	Bacteroides xylanisolvens An107	An107
PRJNA377666	SAMN06473580	Bacteroides xylanisolvens An109	An109
PRJNA377666	SAMN06473581	[Eubacterium] hallii An11	An11
PRJNA377666	SAMN06473584	Flavonifractor plautii An112	An112
PRJNA377666	SAMN06473586	[Clostridium] lactatifermentans An114	An114
PRJNA377666	SAMN06473587	Lactobacillus gallinarum An115	An115
PRJNA377666	SAMN06473588	Alistipes senegalensis An116	An116
PRJNA377666	SAMN06473590	[Eubacterium] contortum An118	An118
PRJNA377666	SAMN06473591	Lactobacillus gallinarum An119	An119
PRJNA377666	SAMN06473592	Drancourtella massiliensis An12	An12
PRJNA377666	SAMN06473593	Gemmiger formicilis An120	An120
PRJNA377666	SAMN06473594	Faecalibacterium prausnitzii An121	An121
PRJNA377666	SAMN06473595	Faecalibacterium prausnitzii An122	An122
PRJNA377666	SAMN06473601	Lactobacillus salivarius An128	An128
PRJNA377666	SAMN06473603	Massiliomicrobiota timonensis An13	An13
PRJNA377666	SAMN06473605	[Eubacterium] fissicatena An131	An131
PRJNA377666	SAMN06473607	Massiliomicrobiota timonensis An134	An134
PRJNA377666	SAMN06473608	Flavonifractor plautii An135	An135
PRJNA377666	SAMN06473610	[Eubacterium] fissicatena An138	An138
PRJNA377666	SAMN06473612	[Clostridium] saccharolyticum An14	An14
PRJNA377666	SAMN06473615	Massiliomicrobiota timonensis An142	An142
PRJNA377666	SAMN06473616	Enterococcus cecorum An144	An144
PRJNA377666	SAMN06473619	[Clostridium] spiroforme An149	An149
PRJNA377666	SAMN06473620	[Clostridium] spiroforme An15	An15
PRJNA377666	SAMN06473623	Lactobacillus gallinarum An153	An153
PRJNA377666	SAMN06473626	[Clostridium] spiroforme An158	An158
PRJNA377666	SAMN06473628	Bacteroides dorei An16	An16
PRJNA377666	SAMN06473630	Bacteroides ovatus An161	An161
PRJNA377666	SAMN06473632	Lactobacillus reuteri An166	An166
PRJNA377666	SAMN06473634	[Clostridium] saccharolyticum An168	An168
PRJNA377666	SAMN06473635	[Clostridium] glycyrrhizinilyticum An169	An169
PRJNA377666	SAMN06473636	Anaeromassilibacillus senegalensis An172	An172
PRJNA377666	SAMN06473637	[Clostridium] spiroforme An173	An173
PRJNA377666	SAMN06473638	Anaerotruncus colihominis An174	An174
PRJNA377666	SAMN06473639	Anaerotruncus colihominis An175	An175
PRJNA377666	SAMN06473640	Pseudoflavonifractor capillosus An176	An176
PRJNA377666	SAMN06473641	Drancourtella massiliensis An177	An177
PRJNA377666	SAMN06473642	[Eubacterium] cylindroides An178	An178
PRJNA377666	SAMN06473643	Butyricicoccus pullicaecorum An179	An179
PRJNA377666	SAMN06473645	Butyricicoccus pullicaecorum An180	An180
PRJNA377666	SAMN06473646	[Clostridium] oroticum An181	An181

PRJNA377666	SAMN06473647	Pseudoflavonifractor capillosus An184	An184
PRJNA377666	SAMN06473648	Clostridium perfringens An185	An185
PRJNA377666	SAMN06473650	Pseudoflavonifractor capillosus An187	An187
PRJNA377666	SAMN06473651	Olsenella uli An188	An188
PRJNA377666	SAMN06473652	Bacteroides clarus An189	An189
PRJNA377666	SAMN06473653	Bacteroides salanitronis An19	An19
PRJNA377666	SAMN06473655	Faecalibacterium prausnitzii An192	An192
PRJNA377666	SAMN06473656	Gemmiger formicilis An194	An194
PRJNA377666	SAMN06473657	[Clostridium] saccharolyticum An196	An196
PRJNA377666	SAMN06473658	Lactobacillus johnsonii An197	An197
PRJNA377666	SAMN06473660	Parabacteroides distasonis An199	An199
PRJNA377666	SAMN06473661	Collinsella tanakaei An2	An2
PRJNA377666	SAMN06473662	Mediterranea massiliensis An20	An20
PRJNA377666	SAMN06473663	Anaeromassilibacillus senegalensis An200	An200
PRJNA377666	SAMN06473664	Anaerofilum agile An201	An201
PRJNA377666	SAMN06473665	Drancourtella massiliensis An210	An210
PRJNA377666	SAMN06473666	Barnesiella viscericola An22	An22
PRJNA377666	SAMN06473667	Cloacibacillus porcorum An23	An23
PRJNA377666	SAMN06473668	Gordonibacter urolithinfaciens strain An230	An230
PRJNA377666	SAMN06473669	Gordonibacter urolithinfaciens An232A	An232A
PRJNA377666	SAMN06473672	Gordonibacter urolithinfaciens An234A	An234A
PRJNA377666	SAMN06473680	Flavonifractor plautii An248	An248
PRJNA377666	SAMN06473681	Blautia schinkii An249	An249
PRJNA377666	SAMN06473682	Anaeromassilibacillus senegalensis An250	An250
PRJNA377666	SAMN06473683	Anaerotruncus colihominis A An251	An251
PRJNA377666	SAMN06473692	[Clostridium] spiroforme An26	An26
PRJNA377666	SAMN06473698	Collinsella intestinalis An268	An268
PRJNA377666	SAMN06473699	Bacteroides salanitronis An269	An269
PRJNA377666	SAMN06473701	Olsenella uli An270	An270
PRJNA377666	SAMN06473702	Collinsella tanakaei An271	An271
PRJNA377666	SAMN06473703	Elusimicrobium minutum An273	An273
PRJNA377666	SAMN06473704	Desulfovibrio desulfuricans An276	An276
PRJNA377666	SAMN06473705	Parabacteroides merdae An277	An277
PRJNA377666	SAMN06473706	Bacteroides salanitronis An279	An279
PRJNA377666	SAMN06473707	Olsenella uli An285	An285
PRJNA377666	SAMN06473708	Megasphaera elsdenii An286	An286
PRJNA377666	SAMN06473709	Muribaculum intestinale An287	An287
PRJNA377666	SAMN06473710	Megamonas hypermegale An288	An288
PRJNA377666	SAMN06473711	Muribaculum intestinale An289	An289
PRJNA377666	SAMN06473712	Olsenella uli An290	An290
PRJNA377666	SAMN06473713	Olsenella uli An293	An293
PRJNA377666	SAMN06473714	[Clostridium] glycyrrhizinilyticum An298	An298
PRJNA377666	SAMN06473715	[Eubacterium] hallii An3	An3
PRJNA377666	SAMN06473716	Flavonifractor plautii An306	An306
PRJNA377666	SAMN06473717	Collinsella intestinalis An307	An307
PRJNA377666	SAMN06473718	Alistipes senegalensis An31A	An31A

PRJNA377666	SAMN06473719	Bacteroides salanitronis An322	An322
PRJNA377666	SAMN06473721	Odoribacter splanchnicus An39	An39
PRJNA377666	SAMN06473722	Flavonifractor plautii An4	An4
PRJNA377666	SAMN06473723	Bacteroides dorei An41	An41
PRJNA377666	SAMN06473724	Parabacteroides johnsonii An42	An42
PRJNA377666	SAMN06473725	Bacteroides clarus An43	An43
PRJNA377666	SAMN06473726	Pseudoflavonifractor capillosus An44	An44
PRJNA377666	SAMN06473727	Odoribacter splanchnicus An45	An45
PRJNA377666	SAMN06473728	Blautia coccoides An46	An46
PRJNA377666	SAMN06473730	Collinsella massiliensis An5	An5
PRJNA377666	SAMN06473731	Gemmiger formicilis An50	An50
PRJNA377666	SAMN06473732	Bacteroides salanitronis An51A	An51A
PRJNA377666	SAMN06473734	Flavonifractor plautii An52	An52
PRJNA377666	SAMN06473737	Alistipes shahii An54	An54
PRJNA377666	SAMN06473738	Barnesiella viscericola An55	An55
PRJNA377666	SAMN06473739	Drancourtella massiliensis An57	An57
PRJNA377666	SAMN06473740	Faecalibacterium prausnitzii An58	An58
PRJNA377666	SAMN06473741	Butyricimonas paravirosa An62	An62
PRJNA377666	SAMN06473742	Lactobacillus salivarius An63	An63
PRJNA377666	SAMN06473743	[Eubacterium] cylindroides An64	An64
PRJNA377666	SAMN06473744	Alistipes senegalensis An66	An66
PRJNA377666	SAMN06473745	Bacteroides uniformis An67	An67
PRJNA377666	SAMN06473746	Clostridium perfringens An68	An68
PRJNA377666	SAMN06473747	Enterococcus cecorum An69	An69
PRJNA377666	SAMN06473748	Collinsella intestinalis An7	An7
PRJNA377666	SAMN06473749	Enorma massiliensis An70	An70
PRJNA377666	SAMN06473750	Lactobacillus reuteri An71	An71
PRJNA377666	SAMN06473754	[Clostridium] lactatifermentans An75	An75
PRJNA377666	SAMN06473755	[Clostridium] glycyrrhizinilyticum An76	An76
PRJNA377666	SAMN06473756	Faecalibacterium prausnitzii An77	An77
PRJNA377666	SAMN06473757	Akkermansia muciniphila An78	An78
PRJNA377666	SAMN06473760	Massiliomicrobiota timonensis An80	An80
PRJNA377666	SAMN06473761	Blautia producta An81	An81
PRJNA377666	SAMN06473762	Flavonifractor plautii An82	An82
PRJNA377666	SAMN06473764	Lactobacillus salivarius An84	An84
PRJNA377666	SAMN06473765	Pseudoflavonifractor capillosus An85	An85
PRJNA377666	SAMN06473766	Gemmiger formicilis An87	An87
PRJNA377666	SAMN06473768	Flavonifractor plautii An9	An9
PRJNA377666	SAMN06473769	Alistipes onderdonkii An90	An90
PRJNA377666	SAMN06473770	Flavonifractor plautii An91	An91
PRJNA377666	SAMN06473771	Flavonifractor plautii An92	An92
PRJNA377666	SAMN14913535	Lactobacillus gallinarum An338a	An338a
PRJNA377666	SAMN14913536	Enorma massiliensis An340a	An340a
PRJNA377666	SAMN14913537	Lactobacillus aviarius An347	An347
PRJNA377666	SAMN14913538	Veillonella magna An354	An354
PRJNA377666	SAMN14913539	Collinsella tanakaei An368	An368

PRJNA377666	SAMN14913540	Pseudoflavonifractor capillosus An386a	An386a
PRJNA377666	SAMN14913541	Clostridium lactatifermentans An390	An390
PRJNA377666	SAMN14913542	Fusobacterium mortiferum An397	An397
PRJNA377666	SAMN14913543	Megamonas hypermegale An400	An400
PRJNA377666	SAMN14913544	Desulfovibrio piger An401	An401
PRJNA377666	SAMN14913545	Bacteroides caecigallinarum An406	An406
PRJNA377666	SAMN14913546	Oscillibacter valericigenes An411	An411
PRJNA377666	SAMN14913547	Desulfovibrio piger An418	An418
PRJNA377666	SAMN14913548	Mordavella massiliensis An420c	An420c
PRJNA377666	SAMN14913549	Mediterranea massiliensis An421	An421
PRJNA377666	SAMN14913550	Faecalicoccus acidiformans An423	An423
PRJNA377666	SAMN14913551	Fusobacterium mortiferum An425	An425
PRJNA377666	SAMN14913552	Bacteroides plebeius An426	An426
PRJNA377666	SAMN14913553	Clostridium glycyrrhizinilyticum An427	An427
PRJNA377666	SAMN14913554	Bacteroides caecigallinarum An428a	An428a
PRJNA377666	SAMN14913555	Bacteroides caecigallinarum An428b	An428b
PRJNA377666	SAMN14913556	Clostridium spiroforme An429	An429
PRJNA377666	SAMN14913557	Clostridium saudiense An430	An430
PRJNA377666	SAMN14913558	Clostridium lactatifermentans An431b	An431b
PRJNA377666	SAMN14913559	Pseudoflavonifractor phocaeensis An431c	An431c
PRJNA377666	SAMN14913560	Clostridium spiroforme An433a	An433a
PRJNA377666	SAMN14913561	Clostridium saudiense An434a	An434a
PRJNA377666	SAMN14913562	Clostridium saudiense An435	An435
PRJNA377666	SAMN14913563	Bacillus licheniformis An441	An441
PRJNA377666	SAMN14913564	Lactobacillus gallinarum An447	An447
PRJNA377666	SAMN14913565	Lactobacillus salivarius An453	An453
PRJNA377666	SAMN14913566	Bacteroides plebeius An475	An475
PRJNA377666	SAMN14913567	Enterococcus cecorum An483	An483
PRJNA377666	SAMN14913568	Pseudoflavonifractor phocaeensis An485	An485
PRJNA377666	SAMN14913569	Bacteroides caecigallinarum An496	An496
PRJNA377666	SAMN14913570	Mordavella massiliensis An499	An499
PRJNA377666	SAMN14913571	Mediterranea massiliensis An502	An502
PRJNA377666	SAMN14913572	Intestinimonas butyriciproducens An503	An503
PRJNA377666	SAMN14913573	Pseudoflavonifractor phocaeensis An507	An507
PRJNA377666	SAMN14913574	Clostridium lactatifermentans An514	An514
PRJNA377666	SAMN14913575	Lactobacillus coleohominis An526	An526
PRJNA377666	SAMN14913576	Veillonella magna An537	An537
PRJNA377666	SAMN14913577	Bacteroides gallinaceum An558	An558
PRJNA377666	SAMN14913578	Phocea massiliensis An559	An559
PRJNA377666	SAMN14913579	Parasutterella secunda An562	An562
PRJNA377666	SAMN14913580	Oscillibacter valericigenes An563	An563
PRJNA377666	SAMN14913581	Hydrogenoanaerobacterium saccharovorans An564	An564
PRJNA377666	SAMN14913582	Intestinimonas butyriciproducens An565	An565
PRJNA377666	SAMN14913583	Clostridium spiroforme An567	An567
PRJNA377666	SAMN14913584	Pseudoflavonifractor phocaeensis An569	An569
PRJNA377666	SAMN14913585	Lactobacillus coleohominis An574	An574

PRJNA377666	SAMN14913586	Gemmiger formicilis An578	An578
PRJNA377666	SAMN14913587	Mordavella massiliensis An582	An582
PRJNA377666	SAMN14913588	Collinsella tanakaei An712	An712
PRJNA377666	SAMN14913589	Collinsella intestinalis An713	An713
PRJNA377666	SAMN14913590	Enorma phocaeensis An718	An718
PRJNA377666	SAMN14913591	Collinsella intestinalis An719	An719
PRJNA377666	SAMN14913592	Gemmiger formicilis An723	An723
PRJNA377666	SAMN14913593	Lactobacillus crispatus An726	An726
PRJNA377666	SAMN14913594	Massiliomicrobiota timonensis An731	An731
PRJNA377666	SAMN14913595	Olsenella uli An732	An732
PRJNA377666	SAMN14913596	Olsenella uli An733	An733
PRJNA377666	SAMN14913597	Eubacterium cylindroides An765	An765
PRJNA377666	SAMN14913598	Streptococcus pleomorphus An766	An766
PRJNA377666	SAMN14913599	Bacteroides coprocola An767	An767
PRJNA377666	SAMN14913600	Bacteroides caecicola An768	An768
PRJNA377666	SAMN14913601	Lactobacillus reuteri An769	An769
PRJNA377666	SAMN14913602	Drancourtella massiliensis An770	An770
PRJNA377666	SAMN14913603	Megasphaera elsdenii An771	An771
PRJNA377666	SAMN14913604	Mediterranea massiliensis An772	An772
PRJNA377666	SAMN14913605	Eubacterium fissicatena An773	An773
PRJNA377666	SAMN14913606	Enterococcus gallinarum An774	An774
PRJNA377666	SAMN14913607	Bifidobacterium saeculare An775	An775
PRJNA377666	SAMN14913608	Megamonas funiformis An776	An776
PRJNA377666	SAMN14913609	Lactobacillus ingluviei An777	An777
PRJNA377666	SAMN14913610	Coprobacillus cateniformis An779	An779
PRJNA377666	SAMN14913611	Clostridium glycyrrhizinilyticum An785	An785
PRJNA377666	SAMN14913612	Escherichia fergusonii An786	An786
PRJNA377666	SAMN14913613	Lactobacillus salivarius An787	An787
PRJNA377666	SAMN14913614	Enorma massiliensis An788	An788
PRJNA377666	SAMN14913615	Collinsella tanakaei An789	An789
PRJNA377666	SAMN14913616	Flavonifractor plautii An790	An790
PRJNA377666	SAMN14913617	Collinsella tanakaei An791	An791
PRJNA377666	SAMN14913618	Collinsella tanakaei An792	An792
PRJNA377666	SAMN14913619	Bacteroides mediterraneensis An793	An793
PRJNA377666	SAMN14913620	Olsenella profusa An794	An794
PRJNA377666	SAMN14913621	Lactobacillus agilis An795	An795
PRJNA377666	SAMN14913622	Lactobacillus agilis An796	An796
PRJNA377666	SAMN14913623	Faecalicoccus pleomorphus An797	An797
PRJNA377666	SAMN14913624	Bacteroides mediterraneensis An801	An801
PRJNA377666	SAMN14913625	Collinsella tanakaei An804	An804
PRJNA377666	SAMN14913626	Megamonas rupellensis An805	An805
PRJNA377666	SAMN14913627	Bacillus cereus An806	An806
PRJNA377666	SAMN14913628	Bifidobacterium stercoris An807	An807
PRJNA377666	SAMN14913629	Clostridium glycyrrhizinilyticum An808	An808
PRJNA377666	SAMN14913630	Megamonas hypermegale An809	An809
PRJNA377666	SAMN14913631	Lactobacillus alvi An810	An810

PRJNA377666	SAMN14913632	Gemmiger formicilis An812	An812
PRJNA377666	SAMN14913633	Lactobacillus salivarius An813	An813
PRJNA377666	SAMN14913634	Fusobacterium mortiferum An814	An814
PRJNA377666	SAMN14913635	Bifidobacterium saeculare An816	An816
PRJNA377666	SAMN14913636	Clostridium saccharolyticum An817	An817
PRJNA377666	SAMN14913637	Bacteroides coprophilus An818	An818
PRJNA377666	SAMN14913638	Marseilla massiliensis An819	An819
PRJNA377666	SAMN14913639	Olsenella uli An820	An820
PRJNA377666	SAMN14913640	Bacteroides gallinaceum An821	An821
PRJNA377666	SAMN14913641	Bacteroides mediterraneensis An822	An822
PRJNA377666	SAMN14913642	Streptococcus alactolyticus An823	An823
PRJNA377666	SAMN14913643	Marseilla massiliensis An824	An824
PRJNA377666	SAMN14913644	Bacteroides gallinaceum An825	An825
PRJNA377666	SAMN14913645	Faecalicatena contorta An826	An826
PRJNA377666	SAMN14913646	Pseudoflavonifractor capillosus An827	An827
PRJNA377666	SAMN14913647	Pseudoflavonifractor phocaeensis An828	An828
PRJNA377666	SAMN14913648	Sutterella massiliensis An829	An829
PRJNA377666	SAMN14913649	Bifidobacterium saeculare An831	An831
PRJNA377666	SAMN14913650	Collinsella tanakaei An833	An833
PRJNA377666	SAMN14913651	Bifidobacterium saeculare An836	An836
PRJNA377666	SAMN14913652	Megasphaera elsdenii An838	An838
PRJNA377666	SAMN14913653	Collinsella intestinalis An840	An840
PRJNA377666	SAMN14913654	Pseudoflavonifractor capillosus An841	An841
PRJNA377666	SAMN14913655	Bifidobacterium saeculare An845	An845
PRJNA377666	SAMN14913656	Eubacterium contortum An846	An846
PRJNA377666	SAMN14913657	Streptococcus pleomorphus An848	An848
PRJNA377666	SAMN14913658	Eubacterium cylindroides An849	An849
PRJNA377666	SAMN14913659	Bacteroides coprocola An850	An850
PRJNA377666	SAMN14913660	Flavonifractor plautii An856	An856
PRJNA377666	SAMN14913661	Megamonas funiformis An949	An949

Supplementary Table S1. List of 259 isolates characterised in this study.

Sample Name	Origin	Environment	Host Age	Host Sex
Exp141_Control_D8_NI_1	Czech Republic	Experimental animal facility	8 days	male
Exp141_Control_D8_NI_2	Czech Republic	Experimental animal facility	8 days	male
Exp141_Control_D8_NI_3	Czech Republic	Experimental animal facility	8 days	male
Exp141_Control_D8_NI_4	Czech Republic	Experimental animal facility	8 days	male
Exp141_Control_D8_NI_5	Czech Republic	Experimental animal facility	8 days	male
Exp142_Control_D8_NI_1	Czech Republic	Experimental animal facility	8 days	male
Exp142_Control_D8_NI_2	Czech Republic	Experimental animal facility	8 days	male
Exp142_Control_D8_NI_3	Czech Republic	Experimental animal facility	8 days	male
Exp142_Control_D8_NI_4	Czech Republic	Experimental animal facility	8 days	male
Exp142_Control_D8_NI_5	Czech Republic	Experimental animal facility	8 days	male
Exp145_Control_D8_NI_1	Czech Republic	Experimental animal facility	8 days	male
Exp145_Control_D8_NI_2	Czech Republic	Experimental animal facility	8 days	male
Exp145_Control_D8_NI_3	Czech Republic	Experimental animal facility	8 days	male
Exp145_Control_D8_NI_4	Czech Republic	Experimental animal facility	8 days	male
Exp145_Control_D8_NI_5	Czech Republic	Experimental animal facility	8 days	male
Exp145_Control_D8_NI_6	Czech Republic	Experimental animal facility	8 days	male
Exp146_Control_D8_NI_1	Czech Republic	Experimental animal facility	8 days	male
Exp146_Control_D8_NI_2	Czech Republic	Experimental animal facility	8 days	male
Exp146_Control_D8_NI_3	Czech Republic	Experimental animal facility	8 days	male
Exp146_Control_D8_NI_4	Czech Republic	Experimental animal facility	8 days	male
Exp146_Control_D8_NI_5	Czech Republic	Experimental animal facility	8 days	male
Exp146_Control_D8_NI_6	Czech Republic	Experimental animal facility	8 days	male
Exp147_Control_D8_NI_1	Czech Republic	Experimental animal facility	8 days	male
Exp147_Control_D8_NI_2	Czech Republic	Experimental animal facility	8 days	male
Exp147_Control_D8_NI_3	Czech Republic	Experimental animal facility	8 days	male
Exp147_Control_D8_NI_4	Czech Republic	Experimental animal facility	8 days	male
Exp147_Control_D8_NI_5	Czech Republic	Experimental animal facility	8 days	male
Exp148_Control_D8_NI_1	Czech Republic	Experimental animal facility	8 days	male
Exp148_Control_D8_NI_2	Czech Republic	Experimental animal facility	8 days	male
Exp148_Control_D8_NI_3	Czech Republic	Experimental animal facility	8 days	male
Exp148_Control_D8_NI_4	Czech Republic	Experimental animal facility	8 days	male
Exp148_Control_D8_NI_5	Czech Republic	Experimental animal facility	8 days	male
Exp149_Control_D8_NI_1	Czech Republic	Experimental animal facility	8 days	male
Exp149_Control_D8_NI_2	Czech Republic	Experimental animal facility	8 days	male
Exp149_Control_D8_NI_3	Czech Republic	Experimental animal facility	8 days	male
Exp149_Control_D8_NI_4	Czech Republic	Experimental animal facility	8 days	male
Exp149_Control_D8_NI_5	Czech Republic	Experimental animal facility	8 days	male
Exp124_Donor_Hen	Czech Republic	Commercial poultry farm	37 weeks	female
Exp130_Donor_Hen	Czech Republic	Commercial poultry farm	34 weeks	female
Exp136_Donor_Hen	Czech Republic	Commercial poultry farm	45 weeks	female
Opat5_Hen	Czech Republic	Commercial poultry farm	45 weeks	female
Drazov_Hen	Czech Republic	Backyard flock	2.5 years	female
Nezam_Hen	Czech Republic	Backyard flock	2.5 years	female
Drazov_Vlcov_Hen	Czech Republic	Backyard flock	2.5 years	female
Vlcov_Hen	Czech Republic	Backyard flock	2.5 years	female

Hannov_W55_4K1	Germany	Commercial poultry farm	55 weeks	female
Hannov_W55_4K2	Germany	Commercial poultry farm	55 weeks	female
Hannov_W55_4K3	Germany	Commercial poultry farm	55 weeks	female
Hannov_W55_4K4	Germany	Commercial poultry farm	55 weeks	female
Hannov_W55_4K6	Germany	Commercial poultry farm	55 weeks	female
Hannov_W55_4K7	Germany	Commercial poultry farm	55 weeks	female
Hannov_W55_4K8	Germany	Commercial poultry farm	55 weeks	female
Hannov_W55_4K9	Germany	Commercial poultry farm	55 weeks	female
Hannov_W55_4P1	Germany	Commercial poultry farm	55 weeks	female
Hannov_W55_4P3	Germany	Commercial poultry farm	55 weeks	female
Hannov_W55_4P4	Germany	Commercial poultry farm	55 weeks	female
Hannov_W55_4P5	Germany	Commercial poultry farm	55 weeks	female
Hannov_W55_4P6	Germany	Commercial poultry farm	55 weeks	female
Mor4_W14_P73	Czech Republic	Commercial poultry farm	14 weeks	female
Mor4_W14_P74	Czech Republic	Commercial poultry farm	14 weeks	female
Mor4_W14_P75	Czech Republic	Commercial poultry farm	14 weeks	female
Mor4_W14_N73	Czech Republic	Commercial poultry farm	14 weeks	female
Mor4_W14_N74	Czech Republic	Commercial poultry farm	14 weeks	female
Mor4_W14_N75	Czech Republic	Commercial poultry farm	14 weeks	female
Mor4_W14_P76	Czech Republic	Commercial poultry farm	14 weeks	female
Mor4_W14_P77	Czech Republic	Commercial poultry farm	14 weeks	female
Mor4_W14_P78	Czech Republic	Commercial poultry farm	14 weeks	female
Mor4_W14_N76	Czech Republic	Commercial poultry farm	14 weeks	female
Mor4_W14_N77	Czech Republic	Commercial poultry farm	14 weeks	female
Mor4_W14_N78	Czech Republic	Commercial poultry farm	14 weeks	female

Supplementary Table S2. List of 70 caecal samples characterised in this study.
